# Athlete Fear Avoidance, Depression, and Anxiety Are Associated with Acute Concussion Symptoms in Athletes

**DOI:** 10.3390/jcm13082401

**Published:** 2024-04-20

**Authors:** Ilana Patlan, Gabrielle Gamelin, Kosar Khalaj, Tristan Castonguay, Geoffrey Dover

**Affiliations:** Department of Health, Kinesiology, and Applied Physiology, Concordia University, Montreal, QC H4B 1R6, Canada; i_patlan@uky.edu (I.P.); gamelingabrielle@gmail.com (G.G.); kosarkhalaj77@gmail.com (K.K.); tristan.castonguay@concordia.ca (T.C.)

**Keywords:** sport, psychosocial factor, pain-related fear, traumatic brain injury, sport-related concussion

## Abstract

**Background:** Assessing sport-related concussions in athletes presents challenges due to symptom variability. This study aimed to explore the relationship between acute concussion symptoms and athlete fear avoidance, pain catastrophizing, depression, and anxiety. Anxiety and depression have previously been associated with the number of symptoms after a concussion, but no prior research has examined the possible link between athlete fear avoidance and acute concussion symptoms. **Methods:** Thirty-four collegiate athletes (mean age = 20.9 ± 1.8 years) were assessed within 48 h of a concussion using the Sport Concussion Assessment Tool 5, Athlete Fear Avoidance Questionnaire (AFAQ), Pain Catastrophizing Scale, and Hospital Anxiety and Depression Scale. **Results:** Results showed a significant association between the athlete fear avoidance and the number of concussion symptoms (r = 0.493, *p* = 0.003), as well as depression and anxiety measured by HADS (r = 0.686, *p* < 0.001). Athlete fear avoidance and HADS scores were predictors of symptom severity, explaining 41% of the variance (*p* = 0.001). Athletes with higher fear avoidance tended to report more symptoms post concussion. **Conclusions:** This study underscores the link between athlete fear avoidance, anxiety, depression, and the severity of concussion symptoms. Administering the AFAQ to assess athlete fear avoidance at the initial assessment of a concussion may be helpful in interpreting the symptoms of an acute concussion.

## 1. Introduction

Concussions are a significant health concern, and a key problem for the assessment of a concussion is the variability in symptoms [1,2,3,4]. In the United States, there are an estimated 3.8 million concussions that occur each year during competitive sports and recreational activities; however, as many as 50% of the concussions go unreported [1]. Although the majority of patients are symptom-free within the first 7–10 days [1,5,6], 10–30% of patients suffer from persisting post-concussion symptoms [5,6]. A sport-related concussion is a clinical diagnosis; however, to date there is no single gold standard diagnostic test [5]. The symptoms commonly associated with a concussion may be linked to the injury but are heterogeneous and not specific enough to be used as the sole assessment test [1,7,8]. For example, symptoms such as headache, nausea, dizziness, and fatigue can be found in healthy non-concussed subjects. The quality and quantity of symptoms suffered by those with a mild traumatic brain injury (mTBI) are similar in other patient populations such as those with persistent pain syndromes [4]. The reason for these large variations in the clinical manifestations of traumatic brain injury (TBI) have been attributed to the complexity of the brain, but no single theory has explained this variability [2].

Psychological factors such as anxiety and depression have previously been associated with the number of symptoms after a concussion [9,10]. Pre-injury mental health was uniquely related to mTBI outcomes in several studies [9,10]. Early post-injury stress and anxiety levels after mTBI are also indicated as predictors of persisting post-concussion syndrome [11,12], and appear to influence the outcome from the mTBI [3]. In a systematic review aimed at improving the Sport Concussion Assessment Tool (SCAT), higher anxiety and depression scores were associated with higher SCAT2 symptom scores in college athletes [8]. Women and adults with early post-injury anxiety also have worse prognoses. However, the severity of mTBI had little impact in the prediction of long-term symptoms [12]. Up to 70% of patients with mTBI report anxiety symptoms, and 21–36% meet diagnostic criteria for anxiety disorder [13,14].

Other psychological constructs such as fear avoidance has been associated with prolonged concussion symptoms. The fear avoidance model (FAM) describes the negative and cyclical nature of the emotional response to pain, including fear of pain, kinesiophobia, fear-avoidance beliefs, and catastrophizing. The fear avoidance model was originally developed to explain the transition from acute pain to chronic pain. In the model, two primary coping reactions to fear of pain exist: confrontation and avoidance. Individuals exhibiting a heightened fear of pain along with fear-avoidance tendencies in response to acute pain are at a greater risk of developing persisting pain compared to those who confront their fear of pain [15,16]. The fear avoidance model has a robust relationship in chronic pain, and there is some evidence that elements of this model could be related to concussions. Previous authors have suggested that there might be a relationship between pain and a fear of re-injury throughout recovery in concussed high school athletes [17]. Another study suggested that patients with a mTBI who had higher levels of fear avoidance suffered from more symptoms. In this study, however, the participants were evaluated on average 48.2 months after their concussion [18]. Since most symptoms resolve in 7–10 days, a more acute assessment of concussions is needed.

The SCAT5 is the gold standard test for the on-field immediate measurement of the concussion [19]; however, as indicated above, the variability in symptoms makes the assessment a challenge [8]. To our knowledge, no previous study has investigated the impact of pain-related fear, catastrophizing, and depression on the acute, on-field measure of concussion within 24–48 h after injury. No prior research has examined the possible link between athlete fear avoidance and acute concussion symptoms. Identifying which athletes present higher levels of fear avoidance may help improve the assessment and tailor the rehabilitation intervention to address both psychological and physical aspects of an injury. The goal of this study was to measure pain-related fear using the Athlete Fear Avoidance Questionnaire (AFAQ), pain catastrophizing using the Pain Catastrophizing Scale (PCS), the Tampa Scale of Kinesiophobia (TSK), and depression and anxiety using the Hospital Anxiety and Depression Scale (HADS), and compare them to the results of the SCAT5 total score and subcomponent scores. We hypothesize that athletes with a greater score on the AFAQ, PCS, TSK, and the HADS will score higher on the SCAT5 total score, and on the symptom subscale of the SCAT5.

## 2. Materials and Methods

### 2.1. Participants

The minimum sample size to achieve 80% power with a 0.05 significance level was calculated to be 18 participants (20% effect size estimation from Dover and Amar, 2015 [20]). We included 34 collegiate athletes (23 male and 11 female) from two colleges and one university. The participants were recruited from various sports including basketball, cheerleading, football, hockey, lacrosse, ringette, rugby, soccer, and volleyball. Criterion sampling was used to recruit potential participants. We included participants who were (1) >18 and (2) sustained a concussion within 48 h of testing. Participants were excluded if they sustained an impact to the head or another part of the body but did not present signs and symptoms of a concussion, or if they were presenting with a concurrent injury. We obtained written informed consent from all participants, and the study was approved by the University Human Research Ethics Committee (#30006430).

### 2.2. Tools

#### 2.2.1. Sport Concussion Assessment Tool 5

We used the SCAT 5 to assess the type of symptoms and the severity of each symptom in the group of concussed athletes. The SCAT5 is a sport concussion assessment tool that can be used by healthcare professionals to evaluate individuals (±13 years of age), who are suspected of having sustained a sports-related concussion [21]. The SCAT (all versions) or its components has been found to present low to moderate levels of bias [7]. We administered the SCAT5 to concussed athletes within 48 h of sustaining a concussion since the diagnostic utility of the SCAT and its components appears to decrease significantly after 3–5 days post injury [8].

The SCAT 5 also includes a balance assessment through the mBESS. Researchers from the University of North Carolina (Chapel Hill, NC, USA) created the Balance Error Scoring System (BESS). It assesses balance through three stances: double, single, and tandem. Athletes are instructed to stand still with hands on hips and eyes closed for 20 s [22]. According to Riemann and Guskiewicz (2000) [23], BESS effectively detects balance discrepancies following a concussion. The modified BESS involves eliminating the unstable surface component and retaining only the three tests conducted on a stable surface [23].

#### 2.2.2. Athlete Fear Avoidance Questionnaire

We used the AFAQ to measure injury-related fear avoidance in athletes [20]. This scale can be used by sports medicine professionals, including athletic therapists and athletic trainers, as an extra rehabilitation tool to identify fear avoidance in athletes as a potential negative psychological barrier to rehabilitation [20]. The AFAQ is shown to have high internal consistency, with an established Cronbach’s alpha coefficient of 0.805 in a study that included 99 varsity athletes [20].

#### 2.2.3. Pain Catastrophizing Scale

We used the PCS to measure pain catastrophizing in concussed athletes [24]. The PCS consists of 13 items measuring the self-reported frequency of catastrophizing thoughts about the experienced pain with a 5-point Likert scale, and includes three subscales: magnification, rumination, and helplessness. The score ranges from 0 to 52, with higher scores indicating a higher intensity of catastrophizing [24]. In apparently healthy individuals, Cronbach’s alpha coefficient for total PCS score was reported as 0.87 [24].

#### 2.2.4. Tampa Scale of Kinesiophobia

We used the TSK to measure concussion-related fear avoidance behavior. This scale initially used to examine kinesiophobia in chronic low back pain patients [25]. The TSK consists of 17 items and uses a 4-point Likert scale with its respective anchors ranging from “strongly disagree” to “strongly agree” [16]. The score on the TSK ranges from 17 to 68, with scores >37 indicating a high level of fear avoidance behavior in patients with pain [16].

#### 2.2.5. Hospital Anxiety and Depression Scale

We used the Hospital Anxiety and Depression Scale (HADS), a common and validated tool for assessing depression in individuals with traumatic brain injury [26]. In addition, this scale can be delivered by any clinician and does not have to be administrated by a psychologist [26]. The HADS is a 14 self-assessment tool with each item reported on a 4-point scale, with 3 indicating higher symptom frequency [26]. The HADS score ranges from 0 to 21, with scores >8 indicating greater levels of depression in patients with traumatic brain injury [26].

#### 2.2.6. 36-Item Short Form

We used the 36-Item Short-Form (SF-36) to estimate pre-injury general health status of subjects [27]. The SF-36 includes one multi-item scale that assesses eight health concepts: (1) limitations in physical activities due to health problems; (2) limitations in social activities due to physical or emotional problems; (3) limitations in usual role activities due to physical health problems; (4) bodily pain; (5) general mental health; (6) limitations in usual role activities due to emotional problems; (7) vitality; and (8) general health perceptions. We used the SF-36—OrthoToolKit (McEck LLC., PA, USA), which is an online calculator to generate a percentage for each of the 8 subscales. We instructed participants to complete the SF-36 based on how they felt prior to the concussion. We acknowledge that using the scale in this manner is not strictly how it was intended, but we wanted to get some idea of the health status of the participant prior to the injury, which was not possible in this study.

### 2.3. Procedures

We approached the Head Athletic Therapists (ATs) from each school and asked them to participate in an information session about our project before the start of the sporting season. The ATs were asked to contact the researchers via phone call or text message when one of their players sustained a concussion. Once the researcher was informed by a team’s AT, the researcher set up a time to meet with the athlete at the clinic. Participants were asked to complete a consent form, after having the researcher explain the goal of the study. During the testing session, a full SCAT5 was completed, and the athlete was asked to fill out the SF-36, AFAQ, PCS, TSK, and the HADS.

### 2.4. Statistical Analysis

All descriptive data was described using mean ± standard deviation (SD). Pearson correlation coefficients (r) were calculated to determine the relationship between the number of concussion symptoms, pain-related fear, anxiety, and depression. Specifically, we identified the relationship between the symptom subcomponent scores of the SCAT5 and all pain-related fear measures (AFAQ, PCS, and TSK, as well as the subscores of the HADS concerning anxiety and depression). We used a multiple linear regression to identify the contribution of AFAQ, HADS anxiety score, and HADS depression score, to the total number of symptoms (dependent variable). A second multiple linear regression was carried out to identify the contribution of each variable, namely, AFAQ, HADS anxiety score, and HADS depression score, to the severity of symptoms (dependent variable). Data analyses were performed using SPSS Statistics 22.0 for Windows 11 (Microsoft, Redmond, WA, USA).

## 3. Results

### 3.1. Participants

**Figure 1:**
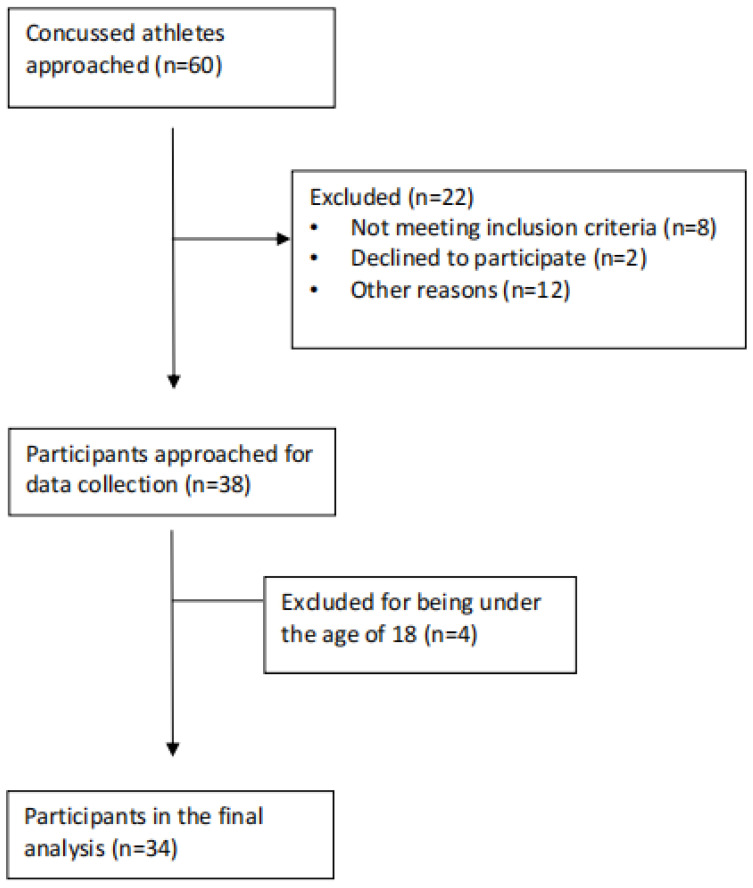
Participant flow through the study.

Approximately 60 athletes were approached to participate in this study after having sustained a concussion. Every athlete included in our study had received a diagnosis of a concussion. Due to travel, however, some athletes could not be assessed within the 48 h time frame. In addition, some athletes chose to go home right after being injured and did not return to school until after the 48 h period. Lastly, some athletes started to participate in the study but could not fill out all the scales required, so they withdrew from the study at that time. As seen in Figure 1, 12 participants found the questionnaires triggering or exacerbating their symptoms, leading them to discontinue their participation in the study (other reasons (*n* = 12)). However, most participants managed to complete the questionnaires by taking breaks, being accommodated in a secluded environment, and receiving assistance as needed, without experiencing a worsening of their symptoms. Therefore, the final number of participant data that were analyzed was 34.

The average age of the participants was 20.9 ± 1.8 years old, with an average height of 178.1 ± 11.1 cm and an average weight of 84.7 ± 25.6 kg. One participant had already been hospitalized for a head injury. Six participants were diagnosed and/or treated for headaches or migraines. To the question “were you ever diagnosed with a learning disability/dyslexia?”, one participant replied with “dyslexia”, and one replied with “verbal dyslexia”, and the rest responded with “no”. To the question “were you diagnosed with attention deficit disorder (ADD)/attention deficit hyperactivity disorder (ADHD)?”, two participants replied with “ADD” and the rest replied “no”. Two participants reported that they were already diagnosed with depression, anxiety, or other psychiatric disorder. Participants were required to be proficient in English and capable of comprehending and completing questionnaires in English. A bilingual (English/French) researcher was consistently available during the data collection period to address any specific clarification questions from participants as needed.

The symptoms and symptom severity are the outcomes of interest and the additional variables included in Table 1 were added to provide a comprehensive overview of all variables collected using the SCAT5.

### 3.2. Relationship between SCAT5 and Psychological Variables

**Figure 2:**
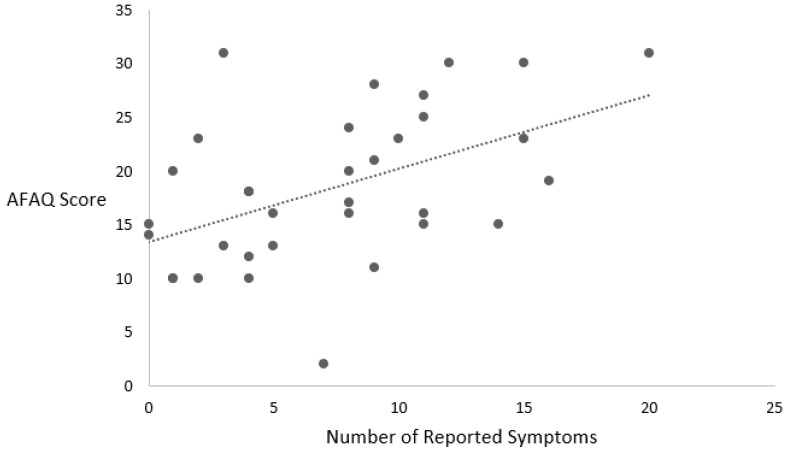
Relationship between number of symptoms and AFAQ score.

The relationship between the SCAT5 and the psychological variables is explained below and listed in Table 2. Figure 2 is used to offer a visual representation of the data distribution.

#### 3.2.1. Total Number of Symptoms and Symptom Severity Score on the SCAT5 and Fear Avoidance

The total number of symptoms reported on the SCAT5 was associated with the AFAQ score (r (32) = 0.493, *p* = 0.003). In addition, the symptom severity score was associated with the AFAQ score (r (32) = 0.481, *p* = 0.004).

#### 3.2.2. Total Number of Symptoms and Symptom Severity Score on the SCAT5 and Anxiety and Depression

The total number of symptoms reported on the SCAT5 was associated with the HADS score (r (32) = 0.686, *p* < 0.001). The symptom severity score was associated with the HADS score (r (32) = 0.602, *p* < 0.001). In addition, the total number of symptoms was associated with both subscales of the HADS including depression (r (32) = 0.614, *p* < 0.001) and anxiety (r (32) = 0.563, *p* = 0.001). Similarly, the symptom severity score was associated with both the HADS depression subscales (r (32) = 0.541, *p* = 0.001) and the HADS anxiety subscales (r (32) = 0.492, *p* = 0.003).

#### 3.2.3. Total Number of Symptoms and Symptom Severity Score on the SCAT5 and Mental Health Pre-Concussion

The total number of symptoms was associated with the mental health subscales of the SF-36 questionnaire (r (32) = −0.571, *p* < 0.001) and the vitality subscales of the SF-36 questionnaire (r (32) = −0.455, *p* = 0.007). The symptom severity score was associated with the mental health subscales of the SF-36 questionnaire (r (32) = −0.518, *p* = 0.002) and the vitality subscales of the SF-36 questionnaire (r (32) = −0.360, *p* = 0.037).

#### 3.2.4. Significant Predictors of Symptom Number and Symptom Severity on the SCAT5

All the variables that were significantly correlated to the number of symptoms and the severity of symptoms were put into two separate linear regressions. The AFAQ score, HADS depression score, and HADS anxiety score model was a significant predictor of the total number of symptoms reported on the SCAT5, accounting for 50.4% of the variance (F (3, 30) = 10.175, *p* < 0.001). The AFAQ score, HADS depression score, and HADS anxiety score model was also a significant predictor of the severity of symptoms reported on the SCAT5, accounting for 41% of the variance (F (3, 30) = 6.936, *p* = 0.001). Table 3 presents the predictor estimates for the symptom severity and symptom count.

## 4. Discussion

### 4.1. Total Number of Symptoms and Symptom Severity Score on the SCAT5 and Pain-Related Fear

Our study identified a significant relationship between athlete fear avoidance, anxiety, and depression with the number of concussion symptoms and the severity of the symptoms within 24 to 48 h of injury. This relationship indicates that athlete fear avoidance may explain some of the variability in concussion symptoms reported in the first 48 h. Previous studies have identified that fear avoidance is associated with more symptoms in persistent concussion symptoms (longer than 3 months) [18], but this is the first study to our knowledge that shows that fear avoidance can influence the number of symptoms in the acute stage (within 24 to 48 h after injury).

Previous studies have identified a relationship between fear avoidance and concussion presentation in persistent concussion symptoms, but the physiological mechanism for this remains unclear. Fear avoidance stems from the belief about the danger in engaging in specific activities or being exposed to certain stimuli, and comes from classic conditioning. The escape and avoidance behavior is the conditioned response which occurs in the anticipation of an unconditioned stimulus, which may be pain or fear or re-injury in the case of concussions [28]. In the first 48 h after a concussion, early attempts to participate in physical or cognitive activity, or being exposed to certain stimuli, may exacerbate certain markers of a concussion. The escalation of symptoms might prompt fear avoidance among athletes, as they may perceive a rise or emergence of new indicators as threatening, thus refraining from engaging in any activity or stimulus that could exacerbate their condition [29]. The latter may explain why an athlete with a high number of symptoms and high severity score may also have a high score on the AFAQ. Although current concussion recommendations suggest an initial period of 48 h of rest before gradually reintegrating physical and cognitive activity [30], initial fear avoidance behaviors may lead to athletes continuing to avoid physical and cognitive activity past this initial rest period.

Fear avoidance has mostly been associated with persistent low back pain. According to the common-sense model, pain-related fear may be a “common-sense” problem-solving response based on a threatening representation of low back pain [31]. The interpretation of pain is led by symptom perception, social messages, and previous experiences related to low back pain. In the absence of a logical explanation of the cause of the pain, behavior will be driven by the emotional response to this pain [31]. Therefore, in the context of a concussion, athletes may not understand the exact cause of the symptoms they are feeling, have had social messages about concussions that may worry them, or have had negative experiences with previous concussions. This may lead to behavior driven by the emotional response to concussion symptoms, which may explain why some athletes may report more concussion symptoms.

The trigger avoidance model of headaches, similar to the fear avoidance model, proposes that avoidance and escape behavior, specific to headache/migraine triggers, may result in increased sensitivity to, and decreased tolerance for, the triggers [32]. In the case of concussions, specifically in the acute stage, athletes do not know the specific activities or stimuli which may cause an increase in symptoms; consequently, an athlete may choose to restrict their activities to avoid the onset and/or severity of symptoms. For example, an athlete may minimize their exposure to light and sound by wearing sunglasses and earplugs during the acute stage of their concussion. This avoidance behavior, however, may make them more sensitive once they return to areas with bright lights or high sound volumes. Therefore, avoidant and escape behavior could also explain why athletes with more concussion symptoms may also have a higher score on the AFAQ.

There have been some previous studies examining concussion presentation and pain catastrophizing [18,33]. One study noted that lingering symptoms and a delay in recovery after a traumatic brain injury (TBI), specifically mTBI, could be explained by the association between post-concussion symptoms and catastrophic thoughts about the symptoms (r = 0.63 in the entire sample and r = 0.69 in the mTBI sample) [18]. Greenberg and colleagues reported pain catastrophizing (b = 0.24, 95% CI) and limiting behaviors (b = 0.14, 95% CI) to partially mediate the relationship between anxiety and post-concussion symptoms after conducting a preliminary simple mediation model in a group of mTBI patients (n = 57) [33]. The authors suggest that to address the lingering concussion symptoms, treating the psychosocial factors may be beneficial. It is important to note that the average time to follow-up with patients in both studies was 48.2 months, which may explain why our symptoms were not associated with pain catastrophizing and may only pertain to persistent symptoms.

During the study, we observed that collecting data on acute concussion symptoms, along with numerous other scales and questionnaires, posed significant challenges for some participants in completing the assessments. It is important to note that requiring concussed participants to engage in extensive assessment tasks is suboptimal, but unfortunately, we lacked a more suitable alternative at the time. In response to these challenges, we ensured that participants were supported in their assessment process. They were encouraged to take all the time they needed to complete the forms, given the opportunity to take breaks, and provided the option to withdraw from the study if they found it too overwhelming.

However, we recognize the need for a more effective approach to symptom measurement that minimizes the exacerbation of participants’ symptoms. While the accommodations offered were helpful, an alternative method, such as digital assessments or non-invasive symptom measurement techniques like imaging or lab testing, could offer a more comfortable and efficient means of data collection for concussed individuals. Incorporating such alternatives alongside existing accommodations could enhance the overall participant experience and data quality in future studies.

### 4.2. Relationship between Anxiety and Depression and Concussion Symptoms and Severity

In a systematic review aimed at improving the SCAT tool, higher anxiety and depression scores were associated with higher SCAT2 symptom scores in college athletes [21]. Similarly to other studies [34], our results also indicated a significant relationship between the number of symptoms and symptom severity in the acute stage of concussion and anxiety and depression. Previously, a combination of neuropsychological, emotional, and traditional measures of severity of head injury taken 7–10 days after injury may help predict post-concussion symptom severity 3 months after injury [35,36]. Similarly, in our study, the symptom severity score was associated with the HADS total score (r (32) = 0.602, *p* < 0.001). Therefore, both studies had similar correlations even though the mean age was different. However, we evaluated athletes within the first 48 h of the concussive impact, while the other study assessed participants 7–10 days post injury.

A study from Ponsford et al. prospectively examined the influence of pre-injury, injury-related, and post-injury psychological factors on post-concussion symptom outcome at 1 week and 3 months post injury [9]. In this study, having had a mTBI (OR 3.30, *p* = 0.001), more anxiety symptoms on the HADS (OR 1.32, *p* = 0.001), and greater pain severity on the visual analog scale (VAS; OR 1.03, *p* = 0.001) were significant predictors of a greater post-concussion score at 1 week post injury. The presence of more anxiety symptoms on the HADS at 1 week was a significant predictor of 3-month post-concussion symptoms (OR 1.18, *p* = 0.001). Premorbid psychiatric factors and post-injury anxiety were the strongest predictors of persistent symptoms at 3 months post injury [9]. In our cross-sectional evaluation within 24–48 h post injury, anxiety was significantly correlated with the total number of symptoms (r (32) = 0.563, *p* = 0.001) and the severity of the symptoms (r (32) = 0.492, *p* = 0.003).

### 4.3. Symptom Level of Concussed Athletes in This Study

Previous studies examined concussion symptoms and severity scores in athletes and head injury patients. Putukian et al. found that collegiate athletes experienced 9.0 ± 5.1 symptoms and severity scores of 19.4 ± 16.9, while our study showed similar results (7.4 ± 5.1 symptoms; 16.3 ± 16.9 severity score) [37]. The consistencies found between the two studies may be explained by the similarities in the population demographics such as age and sport played, as well as the fact that the concussion incidents occurred during a sporting event.

Another study from Bin Zahid et al. on head injury patients reported higher symptom counts and severity scores, likely due to all patients being evaluated in the emergency department [38]. The findings of this study were different from ours because their assessments occurred within the initial 5 days post injury, and none of our participants necessitated hospitalization. This suggests that the severity of our participants’ concussion symptoms may have been less pronounced.

### 4.4. Mental Health Subscore of the SF-36 Questionnaire

We obtained notable findings from our data analysis of the SF-36 questionnaire. The cumulative count of acute concussive symptoms on SCAT5 (r (32) = −0.571, *p* < 0.001) showed an association with the mental health subscore of the SF-36 questionnaire, as did the severity score of symptoms (r (32) = −0.518, *p* = 0.002). This finding implies that forthcoming studies should consider screening for anxiety and depression, as pre-existing mental health correlates with mental well-being during a concussion. Given that mental health factors can contribute to prolonged symptoms post concussion, pre-concussion mental health screening may hold significance. In our study, we asked the participants to fill out the SF-36 based on how they felt before they were concussed. When individuals recall and estimate their premorbid health status, various factors can affect response accuracy, including memory biases and social desirability bias. Therefore, the results should be interpreted with caution as the data cannot be interpreted in the same way as filling out the SF-36 prior to being concussed. It is possible that athletes with a higher mental health subscore on the SF-36 may report more symptoms after being concussed, but this will need to be verified in a future study.

### 4.5. Limitations

One of the limitations of our study is that some of the symptoms suffered by the participants, such as difficulty concentrating, trouble remembering, blurry vision, confusion, sensitivity to light, and feeling slowed down, made it more challenging for participants to fill out the questionnaires. Some athletes had more difficulty reading the questions because of vision difficulties. Some individuals experienced difficulty focusing on the questionnaires, leading to completion times of approximately 30–40 min for both the SCAT5 and other documents. Athletes encountering any challenges should be offered resources to aid them in questionnaire completion to alleviate the burden of reading all the material independently. Another limitation is that not all the questionnaires have been translated to French, and this study was conducted in a province where French is the primary language. Therefore, French-speaking participants reported that certain questionnaires were more challenging than others and required more clarification. Yet, even for English-speaking participants, the wording of certain questions in the questionnaires required clarification.

Finally, another limitation is that this study would have benefited from a control group which would strengthen the conclusions and ideally would have been a group of athletes with orthopedic injuries. Having a control group would have helped identify true concussion symptoms that are independent of the anxious nature of an injury. While previous studies such as those of Dover et al. 2015 and Tito et al. 2023 have noted that elevated athlete fear avoidance has been associated with increased time to return to competition in injured athletes, athlete fear avoidance has not been correlated to non-concussion-specific symptoms [20,39]. This is something that should be considered for future studies.

## 5. Conclusions

One of the challenges in the evaluation of the concussed individual is the variability in symptoms. Our study stands out due to the identification of fear avoidance, depression, and anxiety as predictors of symptom severity in acute concussions. Identifying which athletes present higher levels of fear avoidance may help explain some of the variability in symptoms in concussed athletes, which may improve the accuracy of the assessment. Our results help shed light on the interplay between psychological factors and symptom manifestation post concussion, offering valuable insights into the predictive role of these variables in determining the severity of symptoms experienced by athletes to clinicians. In addition, future studies are needed to examine the possibility of addressing athlete fear avoidance as part of the concussion rehabilitation to minimize symptoms.

According to the fear avoidance model, symptoms are mistakenly interpreted as a sign of serious injury or disease over which one experiences little or no control. This misinterpretation of symptoms may lead to a disproportional fear of these symptoms and injury that, over time, becomes a disabling fear of experiencing symptoms, to the point where these people will avoid participating in these activities for fear that it will make their problem worse [18]. In the acute stage, patients with more athlete fear avoidance may report a higher number of symptoms and a higher severity of symptoms because of this disproportional fear of symptoms and further damage to their brain. Therefore, recognizing fear avoidance, depression, and anxiety as predictors of symptom severity empowers clinicians to develop targeted rehabilitation interventions that address both the physical and psychological aspects of concussion recovery.

## Figures and Tables

**Table 1:** Sample characteristics and SCAT5 results.

	M ± SD or f
Demographics/history	
Sex	
Male (n)	23
Female (n)	11
Age in years (M, SD)	20.9 ± 1.8
Height in cm (M, SD)	178.1 ± 11.1
Weight in kg (M, SD)	84.7 ± 25.6
Sport, (n)	
Basketball	4
Cheerleading	2
Football	7
Hockey	12
Lacrosse	1
Ringuette	1
Rugby	4
Soccer	2
Volleyball	1
Education level (n)	
University	26
College	8
SCAT5 (M, SD) ^†^	
Number of past concussions ^‡^	1.9 ± 1.7
Total number of symptoms ^‡^	7.4 ± 5.1
Symptom severity score ^‡^	16.3 ± 17.0
Hours from injury to assessment ^§^	33.4 ± 17.9
Orientation score (/5)	4.94 ± 0.25
Digits backwards (/4)	3.26 ± 0.93
Months score (/1)	0.90 ± 0.30
Concentration score (/5)	4.19 ± 0.91
Balance (errors/10)	
Double leg	1.65 ± 3.73
Single leg	3.81 ± 2.90
Tandem stance	2.23 ± 3.41

^†^ 5-word and 10-word list score and delayed recall was not reported in results since some used the 5-word list and others used the 10-word list. ^‡^ Number of past concussions, total number of symptoms and symptom severity score were self-reported by the participants on the SCAT 5. ^§^ Hours from injury to assessment were the number of hours from the moment the athlete sustained a head injury to the moment they met with us to complete the questionnaires.

**Table 2:** R-value matrix between variables.

Variable	2. Symptom Severity Score	3. No. of Past Concussions	4. SF-36 Physical Functioning Subscore	5. SF-36 Role Physical Subscore	6. SF-36 Mental Health Subscore	7. SF-36 Vitality Subscore	8. SF-36 General Health Subscore	9. PCS Score	10. TSK Score	11. AFAQ Score	12. HADS Anxiety Score	13. HADS Depression Score	14. HADS Total Score
1. Total no. of symptoms	0.908 **	−0.003	−0.431 *	−0.266	−0.571 **	−0.455 **	−0.228	0.299	0.282	0.493 **	0.563 **	0.614 **	0.686 **
2. Symptom Severity score	-	0.109	−0.374 *	−0.250	−0.518 **	−0.360 *	−0.151	0.33	0.253	0.481 **	0.492 **	0.541 **	0.602 **
3. No. of past concussions		-	0.103	0.074	−0.180	−0.135	−0.148	0.305	0.138	0.332	−0.013	−0.029	−0.26
4. SF-36 Physical Functioning subscore			-	−0.120	0.473 **	0.412 *	0.357 *	0.285	−0.215	−0.504 **	−0.174	−0.626 **	−0.494 **
5. SF-36 Role Physical subscore				-	0.148	−0.048	0.092	−0.096	0.165	−0.228	−0.037	0.024	−0.003
6. SF-36 Mental Health subscore					-	0.798 **	0.493 **	−0.396 *	−0.208	−0.532 **	−0.484 **	−0.418 *	−0.519 **
7. SF-36 Vitality subscore						-	0.452 **	−0.184	−0.156	−0.416 *	−0.307	−0.286	−0.342 *
8. SF-36 General Health subscore							-	−0.138	−0.146	−0.185	−0.170	−0.300	−0.281
9. PCS score								-	0.485 **	0.711 **	0.526 **	0.356 *	0.500 **
10. TSK score									-	0.637 **	0.165	0.366 *	0.321
11. AFAQ score										-	0.346 *	0.475	0.484 **
12. HADS anxiety score											-	0.481 **	0.824 **
13. HADS depression score												-	0.893 **
14. HADS total score													-

* *p* < 0.05. ** *p* < 0.01

**Table 3:** Regression predictor estimates.

Model 1—Concussion Symptoms	Coefficient B	t-Value	Sig	r^2^
Athlete Fear Avoidance	0.152	1.434	0.162	0.504
HADS—Depression	0.521	2.286	0.030 *	
HADS—Anxiety	0.571	2.130	0.041 *	
**Model 2—Symptom Severity**				
Athlete Fear Avoidance	0.592	1.545	0.133	0.410
HADS—Depression	1.411	1.717	0.096	
HADS—Anxiety	1.571	1.624	0.115	

* *p* < 0.05.

## Data Availability

The data presented in this study are available on request from the corresponding author.
